# Improvement of surgical outcomes of complete atrioventricular septal defect with Tetralogy of Fallot

**DOI:** 10.1186/1749-8090-8-S1-P174

**Published:** 2013-09-11

**Authors:** K Morimoto

**Affiliations:** 1 Department of Pediatric Cardiovascular Surgery, National Cerebral and Cardiovascular Center, Suita, Japan

## Background

The life prognoses after the definitive surgery for complete atrioventricular septal defect with tetralogy of Fallot (CAVSD/TOF) have improved recently. However, the reoperation rate for left sided atrioventricular valve (LAVV) regurgitation is still high. We reviewed our surgical experiences for CAVSD/TOF.

## Methods

Between 1981 and 2013, 23 patients with CAVSD/TOF (8 males, median age at operation: 3.0 year old; median weight at operation: 12.0 kg) underwent the definitive surgery: 14 patients before 1995 and 9 patients after 1996. Down syndrome was associated in 17 (74%) patients. The unilateral or bilateral systemic to pulmonary (SP) shunt was placed in 17 (74%) patients, and the timing of the initial SP shunt after 1996 was significantly earlier than that before 1995 (0.1 ± 0.1 vs 4.6 ± 5.8 year old, p= 0.0008). The timing of the definitive surgery after 1996 was also significantly earlier (1.3 ± 0.6 vs 5.7 ± 4.5 year old, p=0.0001). The two-patch repair was applied more frequently after 1996 (p= 0.027).

## Results

The follow-up was completed on all patients and the mean follow-up was 11.3 ± 10.7 years (range, 0.2-32.1). The actuarial survival and freedom from reoperation rates at 30 years were 65.9% and 76.8%, respectively. No mortality and no reoperation have been seen in 10 patients since 1995. Cox’s proportional hazard model revealed that freedom from death or the reoperation rate significantly improved after 1996 when compared to that before 1995 (p= 0.0001). All four reoperations were for LAVV regurgitation. Post-operative LAVV function was better after 1996 (grade of LAVV regurgitation one year after the definitive surgery; 1.5 ± 1.0 vs 2.8 ± 1.3, p=0.04).

## Conclusion

The surgical outcomes of CAVSD/TOF improved over time. The application of two-patch repair, early and frequent palliative SP shunt, and the early definitive surgery may contribute to preserve the post-operative LAVV function.

